# Innate Immune Modulation Induced by EBV Lytic Infection Promotes Endothelial Cell Inflammation and Vascular Injury in Scleroderma

**DOI:** 10.3389/fimmu.2021.651013

**Published:** 2021-04-19

**Authors:** Antonella Farina, Edoardo Rosato, Michael York, Benjamin E. Gewurz, Maria Trojanowska, Giuseppina Alessandra Farina

**Affiliations:** ^1^ Department of Experimental Medicine, Sapienza University, Rome, Italy; ^2^ Department of Clinical Medicine, Sapienza University, Rome, Italy; ^3^ Division of Rheumatology, Boston University School of Medicine, Boston, MA, United States; ^4^ Division of Infectious Diseases, Department of Medicine, Brigham and Women’s Hospital, Harvard Medical School, Boston, MA, United States; ^5^ Program in Virology, Harvard Medical School, Boston, MA, United States; ^6^ Broad Institute of Harvard and MIT, Cambridge, MA, United States

**Keywords:** scleroderma/SSc, Epstein-Barr Virus, endothelial cells, TLR9 innate immune response, vascular injury, digital ulcers, EBV DNA load, monocytes

## Abstract

Microvascular injury is considered an initial event in the pathogenesis of scleroderma and endothelial cells are suspected of being the target of the autoimmune process seen in the disease. EBV has long been proposed as a trigger for autoimmune diseases, including scleroderma. Nevertheless, its contribution to the pathogenic process remains poorly understood. In this study, we report that EBV lytic antigens are detected in scleroderma dermal vessels, suggesting that endothelial cells might represent a target for EBV infection in scleroderma skin. We show that EBV DNA load is remarkably increased in peripheral blood, plasma and circulating monocytes from scleroderma patients compared to healthy EBV carriers, and that monocytes represent the prominent subsets of EBV-infected cells in scleroderma. Given that monocytes have the capacity to adhere to the endothelium, we then investigated whether monocyte-associated EBV could infect primary human endothelial cells. We demonstrated that endothelial cells are infectable by EBV, using human monocytes bound to recombinant EBV as a shuttle, even though cell-free virus failed to infect them. We show that EBV induces activation of TLR9 innate immune response and markers of vascular injury in infected endothelial cells and that up-regulation is associated with the expression of EBV lytic genes in infected cells. EBV innate immune modulation suggests a novel mechanism mediating inflammation, by which EBV triggers endothelial cell and vascular injury in scleroderma. In addition, our data point to up-regulation of EBV DNA loads as potential biomarker in developing vasculopathy in scleroderma. These findings provide the framework for the development of novel therapeutic interventions to shift the scleroderma treatment paradigm towards antiviral therapies.

## Introduction

Systemic sclerosis (Scleroderma, SSc) is a rare heterogeneous autoimmune disease characterized by immune abnormalities, vascular damage and fibrosis (1–4). There is evidence supporting the presence of vascular injury and remodeling in many tissues, including the skin in the early phase of SSc disease, suggesting that endothelial cell injury might be the first pathogenetic event in the development of SSc (4, 5). As a consequence, the progressive vascular dysfunction drives some of the most characteristic clinical features of SSc, including Raynaud’s phenomenon (RP), ischemic digital ulcers, pulmonary arterial hypertension and SSc renal crisis (6, 7). Vascular abnormalities could also precede the onset of fibrosis in the majority of SSc patients, further supporting that endothelial cell dysfunction and microvascular damage might play a key role in the pathogenesis of the disease (5, 8). Despite the importance of the vascular involvement in the pathogenesis of SSc, many aspects of SSc vasculopathy including the nature of the injury and the fate of injured endothelial cells (ECs) remain poorly understood (1, 4, 8).

EBV is commonly associated with autoimmune disorders, including SSc (9–25). One of the most convincing cases is the epidemiological association between EBV seropositivity and two of autoimmune diseases, systemic lupus erythematosus (SLE) and multiple sclerosis (21, 26–28). Importantly, a recent study has demonstrated that EBNA2, an EBV/latent protein colocalizes with autoimmune risk loci in B cells of several autoimmune diseases including SLE, strongly suggesting that EBV contributes to the origin of the genetic risk in these disorders (29). Evidences linking EBV and SSc have been also reported, since EBV latent antigens and high titers of EBV antibodies have been detected more often in SSc patients (30–32). Interestingly, it has been shown that B cells from healthy donor upon EBV transformation were able to produce anti-topoisomerase antibodies (Scl-70) strongly supporting the notion that production of auto-antibodies might be directly related to EBV infection in SSc (33). Despite the extensive range of evidence for a causal link between EBV and autoimmune diseases, to date there is no known mechanism that explains how EBV may contribute to the pathogenesis of these diseases.

The role of EBV`s antigens in triggering autoimmunity has been extensively studied, however, whether the active form of EBV infection (lytic EBV replication) is potentially involved in the pathogenesis of these diseases it is poorly understood (34). Recent evidence suggests EBV lytic reactivation and production of infectious EBV may be pathogenic in several autoimmune disorders, including SSc, while the presence of lytic EBV is found at low levels in healthy populations persistently infected by EBV (10, 12, 35–37). This suggests that viral reactivation occurs more frequently in individuals with a perturbed immune condition (10, 28, 38). In this regard, higher EBV loads in peripheral blood associated with an aberrant serological response to EBV lytic antigens, have been found in patients with autoimmune disorders (30, 34, 39). EBV genome load has been found highly increased in blood from SLE patients, independently of treatment with immunosuppressive agents (9–11) and increased viral activation has been associated with the occurrence of disease activity and flares, supporting the linkage between EBV replication and exacerbation of the disease (10, 40). Recently, we have reported evidence of EBV encoded mRNA and lytic cycle proteins in the majority of fibroblasts and anti-inflammatory (M2) macrophages in SSc (41). We also found that EBV-lytic genes, and proteins were present in SSc monocytes, while small, EBV-encoded RNAs (EBERs) associated with all stages of EBV infection have been detected in dermal ECs of SSc patients, suggesting that monocytes and ECs might represent a target of EBV in SSc (35, 41). We have also demonstrated that the active form of EBV infection drives innate immune inflammation through the induction of the TLR8 inflammatory pathway in infected monocytes, and that SSc monocytes carrying infectious EBV exhibited a robust induction of the IFN signature, as well as altered TLR8 expression compared to healthy donors (HDs) (35). These results showed for the first time that infectious EBV is exclusively present in a subset of SSc monocytes, but not in monocytes from HDs and EBV reactivation triggers a broad spectrum of host genes and cellular pathways including the innate immune responses in the infected cells.

Consistent with our previous finding that SSc monocytes carry EBV active infection and supported by the notion that monocytes have the ability to adhere to the endothelium “*in vivo”* (42), we chose to interrogate the interaction of monocyte-associated EBV with endothelial cells as the mechanism by which the virus induces dysfunctional inflammation and vascular injury in SSc.

In this study, we report that EBV DNA load is remarkably increased in peripheral blood and plasma from SSc patients compared to healthy donor EBV carriers. We also show that B cells and monocytes represent the prominent subsets of EBV-infected cells in SSc, while EBV DNA load is significantly lower or undetected in monocytes from SLE patients as well as in B cells and monocytes from healthy EBV carriers. We demonstrate that human primary dermal endothelial cells are infected with EBV using human monocytes bound to EBV recombinant virus as a cellular shuttle to transfer EBV particles to target cells, while cell-free virus failed to infect the endothelial cells. We show that up-regulation of innate immune mediators, such as TLR9, IRF5, IRF7, IFN-inducible genes MX2 and CXCL10, and several markers of vascular injury are induced by EBV in infected endothelial cells. Finally, to substantiate the association of EBV loads with clinical signs of vascular disease, we show that SSc patients with high level of EBV loads in blood develop an increased number of digital ulcers and marked reduction in skin perfusion, compared to SSc patients with undetectable levels of EBV DNA load with less or no signs of active vascular disease.

Altogether, these results provide new insight into the mechanism employed by EBV in inducing vascular damage through the activation of innate immune inflammatory response and markers of vascular injury in infected endothelial cells. Detection of EBV loads in whole blood and in monocytes can potentially be used as biomarker to evaluate the risk of endothelial cell dysfunction and vascular damage in a cohort of SSc patients with an active EBV infection.

## Materials and Methods

### Study Approval

Experiments were approved by the Institutional Review Board of Sapienza University of Rome (Comitato Etico N° 3377/25-09-14, Rome, Italy) and Boston University (Boston, USA) and performed in accordance with NIH guidelines.

### Study Subjects

All study subjects met the criteria for SSc as defined previously (43). All subjects gave written informed consent. Subjects selected for this study, diffuse cutaneous SSc (SSc) patients: blood (n=65) and skin tissue (n=10), systemic lupus erythematosus (SLE) blood (n=10) and normal healthy donors (HD) blood (n=55) skin tissue (n=10), are summarized in **Supplemental Table 1**. All the patients and HDs included in the study were positive for EBV serology. 71 of 75 SSc patients and 10 SLE patients included in the study were naïve for immunosuppressive therapy (IT) or did not receive any immunosuppressive therapy for a time > 6 months. Four SSc patients were on some form of standard treatment (Mycophenolate or Steroids). Blood samples of these patients were included in the detection of EBV DNA from whole blood. Healthy donors were defined by lacking any current or prior history of malignancy, autoimmune disease, or recurrent/chronic infections. Data such as sex, age, treatment status, disease activity, clinical manifestations, and laboratory parameters were extracted from the medical records of all patients used for these studies and are displayed in **Supplemental Table 1**.

### Monocyte Isolation

Blood was collected from EBV-seropositive HD, SSc and SLE patients in CPT tubes designed for one-step cell separation (Becton Dickinson), and PBMCs were isolated as described previously (44). After positive selection of CD19 cells (CD19+) using magnetic bead isolation (CD19+ selection EasySep, StemCell), monocytes were negatively selected using the Human Monocyte Enrichment Kit without CD16 Depletion (EasySep, StemCell) as described previously.

#### Quantitative PCR to Quantify EBV DNA

DNA was extracted from Whole Blood (WB), plasma and 1–3 × 10^6^ circulating B-cells and Monocytes, using QIAmp DNA blood Mini Kit (Qiagen, Valencia, CA) according to the manufacturers’ protocol. DNA was eluted off the column in an equivalent volume of H_2_0 and stored at -20°C. Previously designed primers and probes that detect a 70 bp region of the EBV BALF5 gene were used (45). EBV DNA was quantified using StepOne™ Real-Time PCR system (Applied Biosystems). Power SYBR Green chemistry (Applied Biosystems) was used for all reactions according to the protocol provided by Applied Biosystems, as described previously. To generate a standard curve, we used an EBV (B95-8 Strain) quantitated viral load control (Advanced Biotechnologies Inc (ABI), Columbia, MD, USA). Each sample was tested in triplicate, and the mean of the two values was shown as the copy number of the sample. The viral loads were log-transformed and then calculated based on EBV genome copies/ml and/or 1–3 × 10^6^ circulating B-cells and Monocytes. The limit of detection by qPCR was 50 copies/mL WB, plasma or per 1-3 X 10^6^ cells.

### Nucleic Acid Extraction, RNA Preparation and Real-Time Polymerase Chain Reaction (q-PCR)

Total RNA from monocytes was extracted using an miRNAsy kit according to the manufacturer`s protocol (Quiagen, Valencia, CA) and processed as reported previously (41). The synthesized cDNAs were used as templates for quantitative real-time PCR. Primer sets designed to detect EBV genes and innate immune mediator genes were used as described previously (35, 41, 46–48). Expression of mRNA for the indicated genes was detected using SYBRGreen chemistry amplification (Applied Biosystems, Life Technologies, Grand Island, NY) as previously described (41). To assure specificity of the primer sets, amplicons generated from the PCR reaction were analyzed for specific melting temperatures by using the melting curve software. All real time-PCR was carried out using StepOnePlus Sequence Detector (Applied Biosystems, Life Technologies, Grand Island, NY). The change in the relative expression of each gene was calculated using δδCt formula choosing the same healthy human subject as the control for all relative expression analyses (47). Target and control reactions were run on separate wells of the same q-PCR plate (47).

#### Tissue Samples

Skin biopsies were obtained from forearm of 10 diffuse SSc patients and 10 healthy donors (HDs). All biopsy specimens were collected under patient consent and approval of the Boston University Medical Campus and “Sapienza” University, Rome, Institutional Review Board. Demographics and clinical characteristics of these cohorts are defined in **Supplemental Table 1**.

#### Histology

All analyses used conventional formalin-fixed, paraffin-embedded sections. Tissue sections were deparaffinised and two-color immunohistochemistry was performed as described previously (35, 49). A double-staining protocol was used on paraffin-embedded slides. After dewaxing, heat antigen retrieval was performerd in Tris/EDTA pH 9.0 for 20 minutes. Blocking was achieved using 3% H_2_O_2_ followed by BloxAll (Vector Labs, Burlingame, CA) or 2% horse serum. Antibodies (Abs) were separately titrated for the two modalities. Primary Abs were mouse anti-human CD31 (mAbs C31.3 + JC/70A; Novus Biologicals, Littleton, CO), mouse anti-Zebra mAb (AZ69, Argene Varilhes, France). Appropriate Vector ImmPress Polymers (ImmPress AP or ImmPress P anti-mouse IgG Polymerdetection kit) were used to detect primary antibodies, followed by development with either HighDef Blue (Enzo,Farmingdale, NY, USA, alkaline phosphatase), or DAB (DakoCytomation) [brown, horseradish peroxidase (HRP)].

### Tissue Culture

Human dermal microvascular endothelial cells (HDMECs, ECs) were isolated from foreskin biopsies, grown on collagen-coated 2 well glass chamber slides in Endothelial Cell Basal Medium-2 (EBM-2) (Lonza, Walkersville, MD) and characterized as described previously (50). EBV-p2089 is a recombinant virus, generated by inserting EBV (B95-8) genome into a Bacterial Artificial Chromosome (BAC) and produced in a cell line (2089/293) stably transduced with genes for hygromycin resistance and green fluorescent protein (GFP) under selection (kind gift of Dr. Henri-Jacques Delecluse, German Cancer Research Center, Heidelberg, Germany) as described previously (51, 52). All cells were cultured in the presence of penicillin/streptomycin and fetal bovine serum. For continuous culture of EBV-p2089/293, hygromycin *(*100 μg/ml) were added to the cultures.

### Monocyte-Endothelial Cells Transfer Infection

*Virus preparations:* preparations were made from 293 cells carrying recombinant B95.8 EBV genomes (EBV/p2089), as previously described (51–53). Briefly, 293 cells carrying EBV/p2089 were transfected with wt-Zta plasmid (pCMV-Zta) (kind gift of Dr. George Miller, Yale University) to stimulate virus production (54). After 6 days post transfection, supernatants were collected and EBV-p2089 concentration quantitated by qPCR. EBV genome was also assayed by immunofluorescence as reported previously (51–53, 55).

*Monocyte EBV/p2089 binding assay:* freshly isolated monocytes from healthy donors (10^6^ cells/mL) were irradiated with high dose of UV (2500 µjoule/cm^2^). Subsequently, 10^5^ UV irradiated monocytes were re-suspended in virus preparations (500000 virus DNA copies/mL in RPMI medium 1640) (EBV/p2089 loaded monocytes) or in medium without virus preparations (mock loaded monocytes) for 4 hours (hrs) at 4°C. Cell viability was evaluated in EBV/p2089 loaded monocytes and mock loaded monocytes at baseline and 4-48 hrs post UV-irradiation treatment by trypan blue dye exclusion procedure. EBV/p2089 binding to monocyte surfaces was quantitated by qPCR amplifying within the BALF5 gene.

*Transfer infection:* 10^5^ virus loaded monocytes or mock loaded monocytes were added to 0.5/mL wells that had been seeded 48 hrs earlier with 3 x 10^5^ endothelial cells. Each transfer infection or mock infection has been made in triplicates and each experiment was repeated five times with different monocyte healthy donors and endothelial cells lines. After co-culture for up to 24 hrs, supernatants were removed from endothelial cells cultures by washing and replaced with fresh medium for 24 hrs. Transfer infection was assayed at 24 and 48 hrs after the initiation of co-culture by counting the percentage of GFP-positive cells in the cultures. After co-culture for up to 48 hrs post infection (PI), cells were harvested and processed for DNA, RNA and immunostaining analysis. Viral genome in endothelial cells was quantitated by qPCR with a BALF5 gene probe. EBV/p2089-GFP in endothelial cell cultures was detected using a FluoView FV10i confocal microscope system (Olympus, Center Valley, PA) at 488 (green) and 405 nm (blue). Original magnification 60x.

### Immunocytochemistry (ICC) and Immunofluorescence (IF)

For ICC analysis, cells were grown on Nunc glass 2 well chamber slides, fixed and permeabilized with ice-cold acetone/methanol (1:1) at -20°C for 3`. No heat antigen retrieval was performed. Blocking was achieved using 3% BSA. A double immunocytostaining was performed after blocking and endothelial cells stained with rabbit anti-human von-Willebrand-Factor (VWF) polyclonal abs (DakoCytomation), mouse anti-Zebra mAb as described above and mouse anti-human CD163 mAb (EDHu-1, AbD Serotec, Raleigh, NC). Appropriate Vector ImmPress Polymers (rabbit, and mouse) were used to detect primary antibodies, followed by development with either Vector AMEC [red, horseradish peroxidase (HRP)], HighDef Blue (Enzo, HRP or alkaline phosphatase), or DAB (DakoCytomation) [brown, horseradish peroxidase (HRP)].

For IF, cells were fixed with 100% acetone. ECs were stained sequentially with mouse anti-LMP1 monoclonal abs (CS.1-4, DakoCytomation), Cy3-conjugated-labeled donkey anti-mouse IgG, quenching with mouse IgG (Jackson IR, West Grove, PA), followed by counterstaining with DAPI (Vectashield, Vector Laboratories, Burlingame, CA). Original magnification as indicated.

### Nailfold Videocapilloscopy (NVC)

NVC was performed with a videocapillaroscope (Pinnacle Studio Version 8) equipped with a 500 × optical probe. Based on Cutolo et al., ‘SSc patterns’ were described as early, active and late (56). At the same time, whole blood was collected from SSc patients and EBV DNA extracted as described above.

### Laser Speckle Contrast Analysis (LASCA)

LASCA was performed after resting the subject in a temperature-controlled room at 24 ± 1°C for 20 min. According to previous studies, peripheral blood perfusion (PBP) of hand dorsum was measured by LASCA (Pericam, Perimed, Sweden). The scanner was placed perpendicularly 15 cm away from the hands according to the manufacturer’s instructions. Two-dimensional images (measurement area 12 × 12 cm) were acquired at the highest time and spatial resolution. PBP was expressed by arbitrary perfusion units (pU). All values are calculated as mean of both hands (57, 58).

### Statistical Analysis

For qPCR EBV DNA quantification and mRNA expression results data are expressed as the mean ± SEM. Statistical comparisons between groups were tested by two-tailed *t* test. Significance was taken at *P ≤* 0.05. For the NVC and LASCA the results are expressed as median and interquartile range (IQR). SPSS version 25.0 software was used for statistical analysis. The tShapiro–Wilk test was used to evaluate normal distribution of data. Group comparisons were made by Mann-Whitney test. Spearman’s rank correlation coefficient was used to test for associations between numerical variables. The chi-square test or Fisher’s exact test, as appropriate, were used to compare categorical variables. P-values < 0.05 were considered significant.

## Results

### EBV DNA Loads Is Increased in Multiple Blood Components of Patients With Scleroderma

**Figure 1:**
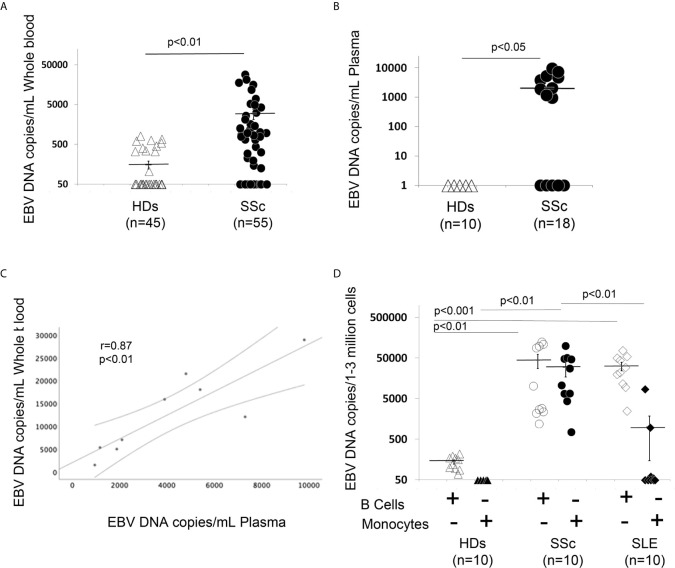
EBV DNA load is increased in blood, plasma, B cells and monocytes from patients with SSc. **(A, B)** EBV DNA was extracted from whole blood **(A)** or plasma **(B)**. **(C)** SSc patients with high copies of EBV DNA in blood show high copies of EBV in plasma (Spearman r=0.81, p<0.01). **(D)** EBV DNA was extracted from B cells and monocytes. EBV DNA was quantified by qPCR. EBV quantitative curve was generated using ABI`s Viral load control (Advanced Biotechnologies, Columbia, MD, USA). qPCR analysis was performed using primers and probe designated to amplify the EBV polymerase BALF5, and measured using SYBR Green chemistry. Shown here are copies of viral DNA calculated by standard curve. All the samples were tested in triplicates. The average of copies number is represented by horizontal line ± SE. p-values calculated using Student T-Test 2 tails.

Evidence of high EBV viral loads have been reported in peripheral blood from patients with autoimmune diseases (30, 34, 39). Specifically, EBV lytic genes in B cells from patients with SLE and increased viral activation have been associated with the occurrence of disease activity and flares in these patients (10, 40). Association of higher levels of cell-associated viral genomes in circulating blood cells have also been found in patients with rheumatoid arthritis (RA), where it has been shown that viral replication correlated with enhanced EBV-specific immune responses in RA, further supporting the linkage between EBV replication and exacerbation of autoimmune diseases (10, 12, 40). Supported by the finding that EBV replication occurs in SSc (35), we sought to quantify circulating EBV DNA load in SSc patients. EBV DNA load was significantly increased in 30 of 50 (60%) SSc patients, while it was detectable in 11 of 45 (24%) healthy donor (HD) EBV carriers in whole blood (WB). Mean EBV DNA copies/mL were 2973.3 for SSc vs 154 in HDs 1mL of WB (**Figure 1A**). We also found significantly high levels of EBV loads in plasma from 9 SSc patients (50%), while EBV was undetected in plasma from all HD EBV carriers (**Figure 1B**). Interestingly, EBV loads in WB and those in plasma correlate with each other to some extent, though viral DNA was detectable in WB, but not in plasma in some patients (**Figure 1C**).

Given that EBV DNA can exist in different forms, such as cell-free EBV-DNA in plasma or cell-associated EBV in WB, we next investigated whether monocytes and/or B cells might also carry the viral genome in SSc. 10 SSc patients with active diffuse cutaneous disease and 10 HD EBV carriers were selected to quantify the amount of EBV DNA load in freshly isolated B cells and monocytes. We found strikingly increased EBV DNA loads in monocytes and B cells from SSc patients, but not in monocytes from healthy EBV carriers (**Figure 1D**). We also found a low viral DNA level in B cells from HDs, likely from latent EBV infection. Given that EBV loads are increased in SLE patients, a disease with overlapping autoantibody specificities and sometimes overlapping clinical manifestations with SSc (59–61), we sought to quantify the amount of cellular-EBV in B cells and monocytes from SLE patients. We found that EBV DNA load is significantly increased in SLE B cells compared to the HDs, while a slightly increased EBV DNA load was detected in monocytes from 3 (30%) SLE patients (**Figure 1D**). These results suggest that monocytes might be a specific target of EBV replication in SSc, but not in patients with SLE.

### ZTA/EBV Lytic Antigen Is Expressed in the Vessels of SSc Skin

**Figure 2:**
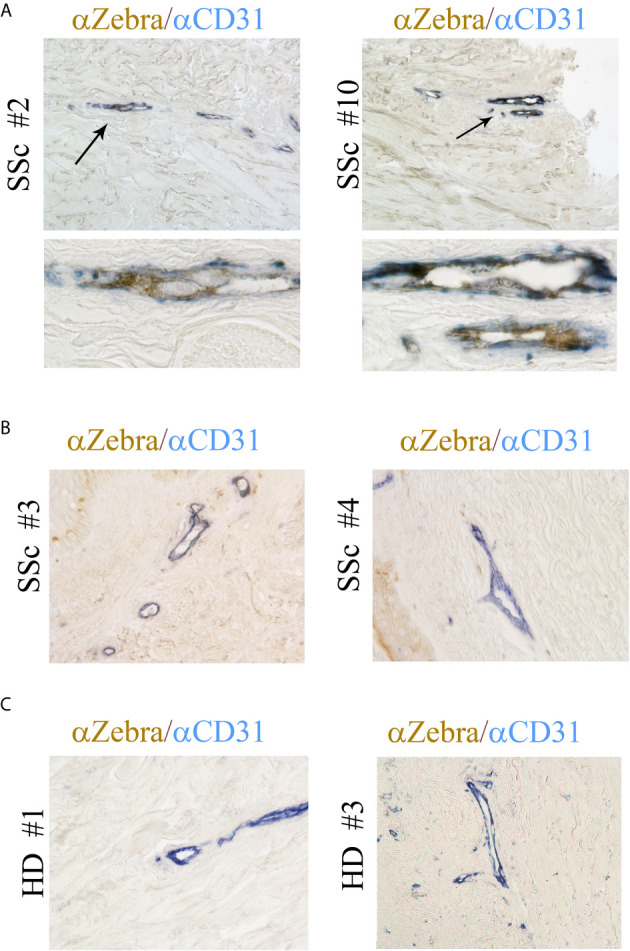
ZTA/Zebra-EBV lytic antigen is expressed in the vessels from SSc. **(A–C)** Dual Immunostaining for ZTA/EBV (brown) and CD31 endothelial cell antigen (blue) in skin sections from four representatives SSc patients and two representative healthy donors. **(A)** Arrows indicate magnification of double positive staining in SSc biopsies. Upper image original magnification 100x; lower image: magnification 1000x. **(B, C)** Original magnification 100x.

Our previous study showed that EBV RNAs, mostly represented by EBERs are also present in the endothelial cells in the skin of SSc patients (41). To further explore whether lytic EBV antigens are present in dermal ECs, the abundance of EBV-encoded immediate early lytic transcription factor ZTA (also called Zebra or BZLF1) was evaluated in the vessels from SSc and HDs skin biopsies. Dermal vessels were identified using the CD31 marker, a specific antigen for ECs. Immunohistochemical staining of SSc skin showed that ZTA/EBV+ cells co-localized with CD31+ cells in two of ten SSc skin biopsies (20%), while it was undetected in CD31+ endothelial cells from eight SSc and ten HD skin samples (**Figures 2A–C**). Interestingly, ZTA staining was mostly detected in damaged or apoptotic cells of the vessels that appeared to be destroyed (**Figure 2A**, lower images), suggesting that EBV lytic-infection damages nuclei in the infected cells (62). Zta positive cells and negative for CD31 antigen were also detected in eight of ten SSc skin samples (**Figure 2B**), indicating that non-endothelial cells, possibly fibroblasts and monocytes, were infected with EBV, as we reported previously (35, 41).

### EBV Infects Human Dermal Microvascular Endothelial Cells *In Vitro*

**Figure 3:**
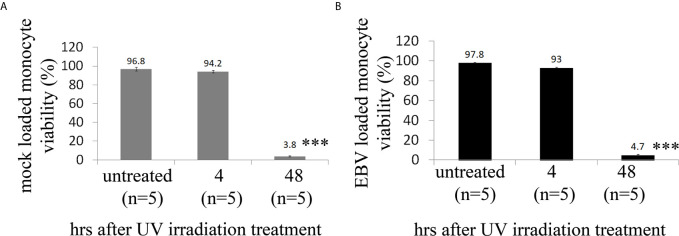
Cell viability of human monocytes after exposure of UV irradiation treatment. **(A, B)** Freshly isolated human monocytes from healthy donors (HDs) were irradiated with high dose of UV and then re-suspended in virus preparations (EBV/p2089 loaded monocytes) or in medium without virus preparations (mock loaded monocytes). Cell viability was evaluated by trypan blue dye exclusion procedure. Bars represent mean ± S.E.M. from five different HDs. p-values (p<0.0001) calculated using two-tailed T-test. ***p<0.0001.

**Figure 4:**
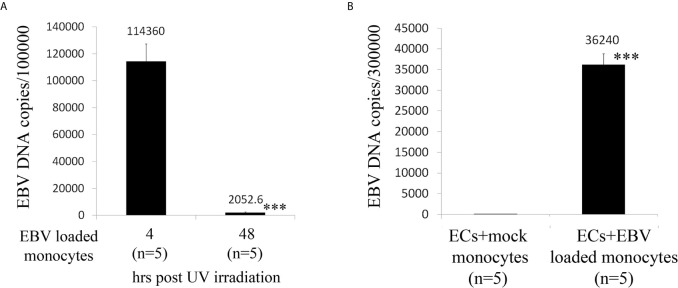
Assay for virus genomic copies in monocytes and endothelial cells. **(A)** Irradiated monocytes were exposed to EBV preparations at known concentration (EBV/p2089 loaded monocytes) or re-suspended in medium without virus preparation (mock loaded monocytes). **(B)** EBV/p2089 loaded monocytes and mock loaded monocytes were co-cultured with human dermal microvascular endothelial cells (HDMECs) grown on collagen-coated chamber slides. After co-culture for up to 48 hrs post infection (PI), cells were harvested and processed for DNA assay. **(A, B)** Virus DNA copies per exposed cells were quantified by qPCR amplifying within the BALF5 gene and measured using SYBR Green chemistry. Shown here are copies of viral DNA calculated by standard curve. All the samples were tested in triplicates. The average of copies number is represented by horizontal line ± SE. p-values calculated using Student T-Test 2 tails. ***p<0.0001.

**Figure 5:**
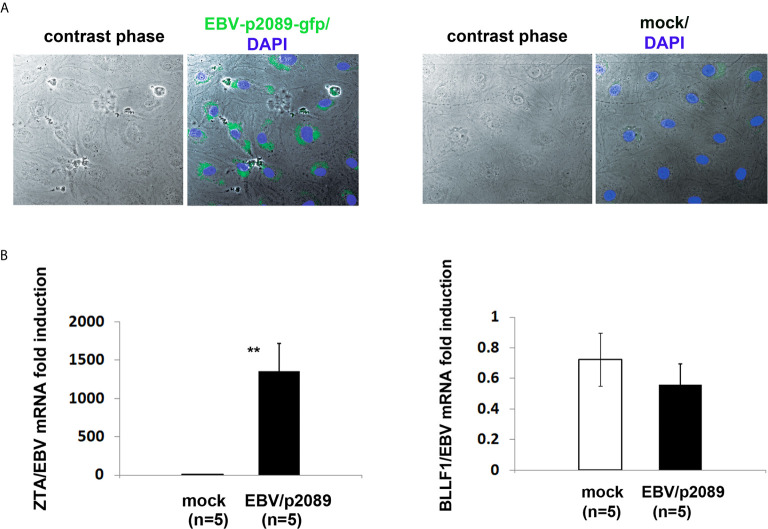
EBV/p2089 infection of human dermal microvascular endothelial cells *in vitro.* Monocytes from healthy donors previously exposed to EBV-p2089 were co-cultured with human dermal microvascular endothelial cells (HDMECs) grown on collagen-coated chamber slides. In parallel, mock infected cultures were established by co-culturing HDMECs with monocytes not exposed to EBV-p2089. After 48h monocytes and EBV-p2089 free virus were removed from endothelial cells. **(A)** One representative of five experiments. Immunofluorescence staining of HDMECs infected with EBV-p2089. Cells were fixed with 100% acetone and mounted using Vectashield mounting medium with DAPI. EBV-p2089-GFP in endothelial cell cultures was detected using a FluoView FV10i confocal microscope system at 488 (green) and 405 nm (blue). Original magnification 600x. **(B)** Expression of EBV lytic genes by qPCR in EBV-p2089 infected endothelial cells. Data are expressed as the fold-change normalized to mRNA expression in a single sample of mock infection. Bars represent mean ± S.E.M. **p<0.001.

As ECs are negative for the EBV CD21/CR2 receptor employed by EBV to infect B cells and T-cells (63–65), we used monocytes bound to recombinant EBV/p2089 (EBV/p2089 loaded monocytes) as the vehicle to infect human dermal primary microvascular endothelial cells (HDMECs) (EBV-monocyte transfer-infection) (55, 66, 67), as previously shown in fibroblasts (41). EBV/p2089 is a recombinant virus equipped with the full spectrum of viral infection programs that allows efficient infection of various primary human cells and a green fluorescence protein (GFP) marker (51, 52, 67). Based on the EBV/p2089 encoded GFP marker, this system provides an efficient way to track recombinant EBV infection and EBV genome dependent gene expression in infected cells. Given that monocytes might persist longer in most cellular cultures, potentially contaminating endothelial cell purity, lethally irradiated cells have been used as vehicle to infect ECs with EBV (66), Therefore, we pretreated monocytes with UV irradiation before exposing them to EBV/p2089 preparations. Viability of EBV/p2089 loaded monocytes and mock loaded monocytes was less than 5% in both condition (**Figures 3A, B**), suggesting that almost all monocytes die in cultures 48 hrs post UV irradiation treatment. Monocytes not exposed to UV treatment survived 7 days in cultures (data not shown). After exposing irradiated monocytes to EBV/p2089, we next asked whether virus binding to monocytes reaches quantifiable levels in irradiated cells. Viral genome load was highly increased in virus loaded monocytes (**Figure 4A**), suggesting that EBV/p2089 is capable of efficiently binding to irradiated monocytes. We then posed the question whether virus loaded monocytes, even though dying, remain capable of mediating virus transfer to endothelial cells. Significantly increased load of EBV/p2089 was detected in infected endothelial cells (**Figure 4B**) and EBV/p2089-GFP-fluorescent signal was localized in infected cells (**Figure 5**). An estimated 40% ECs showed perinuclear and cytoplasmic/cytoskeletal GFP-fluorescence at 48 hours post infection (PI) (**Figure 5A**). Overall, these results suggest that efficient rates of infection can be achieved by using virus loaded irradiated-monocytes in mediating transfer infection to endothelial cells.

**Figure 6:**
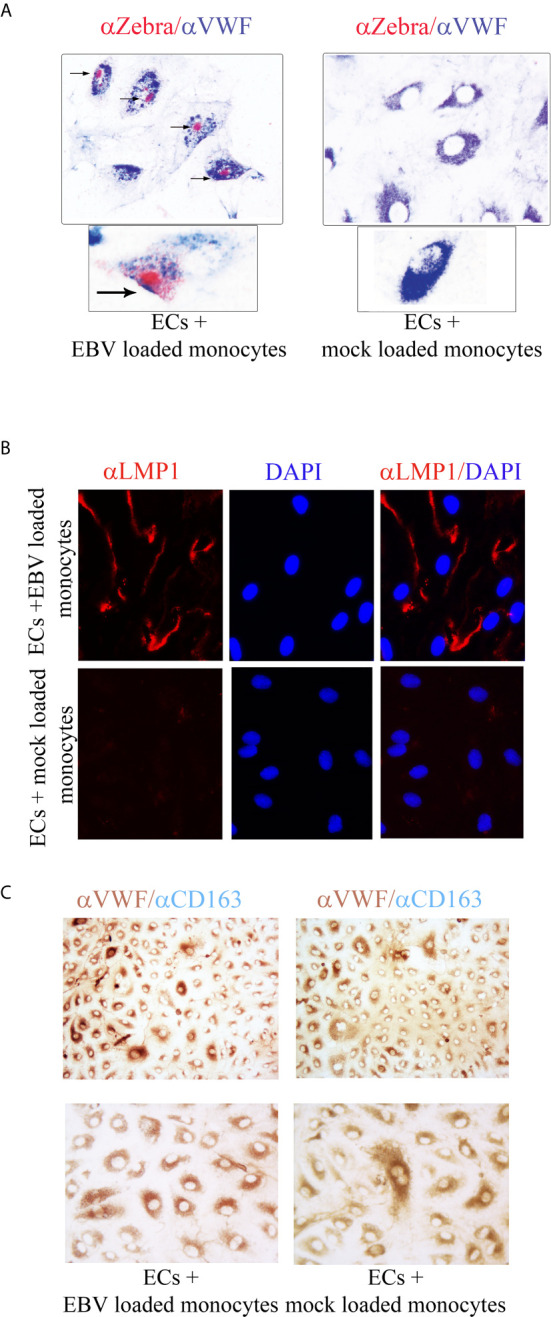
ZTA/EBV lytic and LMP1/EBV antigens are expressed in human dermal microvascular endothelial cells infected with EBVp2089. EBV/p2089 loaded monocytes were co-cultured with endothelial cells. After 48 hrs monocytes and free cell EBV-p2089 were removed. **(A)** Dual immunocytochemistry (ICC) staining showing Von Willebrand Factor (VWF) antigen in the cytoplasm and Zta protein in the nuclei of a subset of infected endothelial cells. Original magnification 400x, box area: original magnification 1000x. **(B)** Immunofluorescence of endothelial cells stained with LMP1/EBV latent antigen (red) as indicated. Diaminidino-2-phenylindole (DAPI) was used as counterstaining for the nuclei. Original magnification 400x. **(C)** Dual ICC staining showing positive cells for Von Willebrand Factor (VWF) antigen in the cytoplasm (brown) and negative for CD163 mononcyte surface antigen (blue). Upper image Original magnification 100x, lower image original magnification 400x. Representative of 5 experiments.

To further characterize the EBV infection program in endothelial cells, we measured expression of latency, immediate early, early, and late lytic-genes were tested. We found expression of ZTA in a population of EBV/p2089 infected cells positive for Von Willebrand Factor (VWF), an endothelial cell antigen, while EBV/late lytic gene BLLF1 which encodes gp350 was not detected (**Figure 5B** and **Figure 6A**). These results suggest that EBV replication was abortive in ECs. Expression of EBV Latent Membrane Protein 1 (LMP1) was also detected in a distinct population of ECs, indicating that EBV may establish latent infection in a subset of ECs (**Figure 6B**), though LMP1 can also be expressed as a lytic antigen (68, 69).

To further evaluate the purity of the EC population after EBV/p2089-monocyte transfer, monocyte markers were evaluated by qPCR, using mRNA extracted from EC cultures. CD14 and CD163 mRNA expression were undetectable in mock infected and monocyte transfer-infected-endothelial cells (data not shown). Accordingly, we did not detect CD163 monocyte surface marker expression in the endothelial cell cultures, further confirming the absence of monocytes in these cultures (**Figure 6C**). Cell free EBV/p2089-virus failed to infect endothelial cell lines.

### EBV Induces TLR9 Innate Immune Responses and Markers of Vascular Injury in Infected ECs

**Figure 7:**
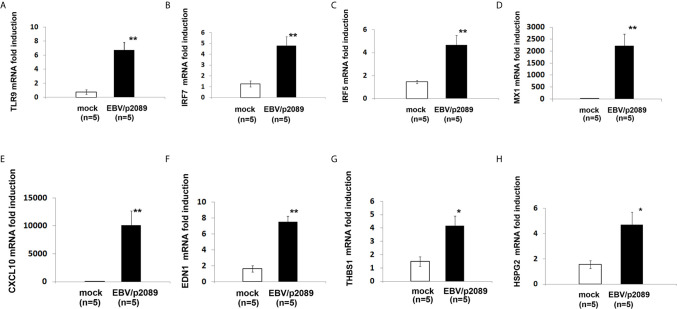
EBV activates innate antiviral response and markers of vascular injury in infected endothelial cells. Monocytes from healthy donors (HDs) bound to EBV-p2089 were co-cultured with human dermal microvascular endothelial cells (HDMECs). Monocytes not exposed to EBV-p2089 were co-cultured with HDMECs as mock-infection. After 42h monocytes EBV-p2089 cell-free virus were removed from endothelial cell cultures and total RNA extracted after. **(A–H)** mRNA expression of indicated genes in EBVp2089-infected and mock-infected endothelial cells, evaluated by qPCR. Fold-changes shown on the graph are normalized to mRNA expression by one mock infected cell line. Bars represent mean ± S.E.M. from 5 separate endothelial cell lines. p-values calculated using two-tailed T-test. *p<0.05, **p<0.001.

While evidence supports activation of the innate immune response in mediating inflammation in SSc (2, 70, 71), the mechanisms by which the immune deregulation can affect the endothelium in SSc is still unclear. Given the prominent contribution of EBV lytic infection in inducing activation of the innate immune response in infected monocytes and fibroblasts (35, 41), we sought to evaluate whether EBV might also induce a similar innate immune response in EBV/p2089 infected endothelial cells. Based on the previous reports describing pro-inflammatory genes, IFNα and markers of vascular inflammation such as ET-1 mRNA (EDN1) as increased in SSc vessels and skin (46, 72), we choose to assess the expression of these genes in endothelial cells infected with EBV/p2089- compared to mock infected cells. Expression of TLR9 mRNA was significantly induced in EBV/p2089-infected-ECs (**Figure 7A**), as were mRNAs encoding IRF7, IRF5 and selected Interferon-stimulated-genes (ISGs), such as MX1 and CXCL10 (**Figures 7B–E**). TLR3, TLR4 and TLR7 mRNA expression was not detected in EBV-p2089 infected cells (data not shown), suggesting that EBV-induced innate immune response is mediated by TLR9 in infected ECs. Interestingly, genes which were previously identified as markers of vascular dysfunction in SSc, such as EDN1, thrombospondin 1 (THBS1), and heparan sulfate proteoglycan 2 (HSPG2) (46, 72, 73) were also induced by EBV in infected endothelial cells (**Figures 7F–H**). No increase of TLRs, IRFs or ISGs was observed in mock infected endothelial cell cultures (**Figures 7A–H**). Altogether, these results suggest that EBV induces activation of the TLR9 innate immune response in infected endothelial cells possibly contributing to endothelial cell dysfunctional activation and injury in SSc.

### Increased EBV DNA Load Correlates With Clinical Patterns of Vascular Injury in SSc Patients

**Figure 8:**
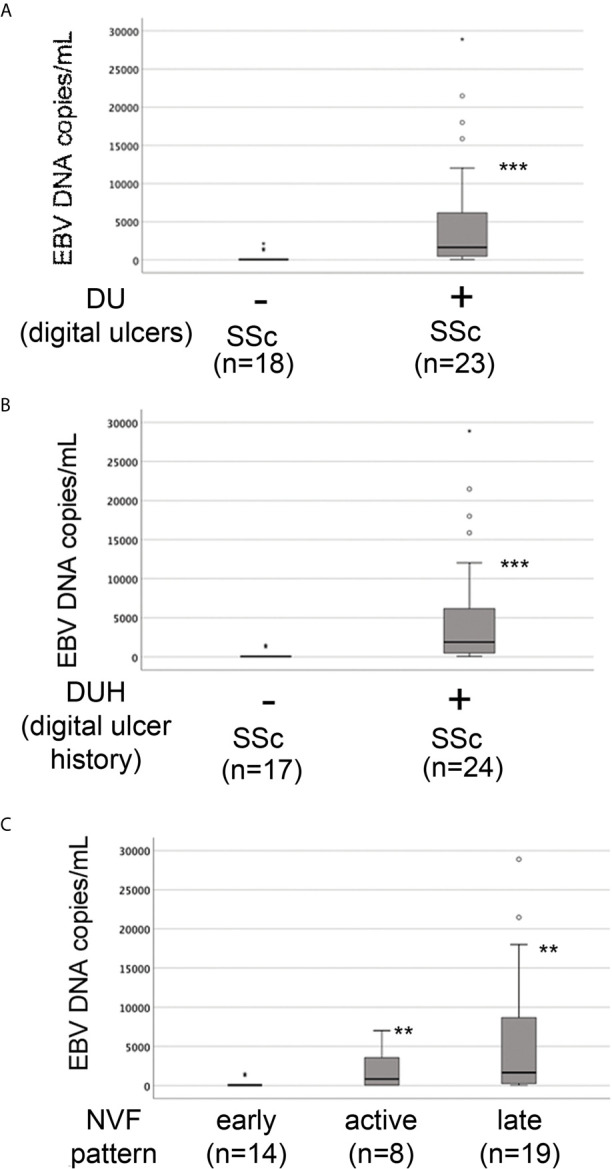
Increased EBV loads correlate with an increased number of digital ulcers and patterns of microvascular damage in SSc. EBV DNA was extracted from whole blood and quantified by qPCR. EBV quantitative curve was generated using ABI`s Viral load control. qPCR analysis was performed using primers and probe designated to amplified the EBV polymerase BALF5, and measured using SYBR Green chemistry. Shown here are copies of viral DNA calculated by standard curve. **(A, B)** SSc patients with active digital ulcers and past history of digital ulcers showing higher level of EBV DNA loads compared to SSc patients with no or fewer digital ulcers showing low or undetectable level of EBV DNA loads. **(C)** SSc patients with increased level of EBV DNA loads show active and late patterns detected by nailfold videocapillaroscopic compared to patients with low or undetectable level of viral loads in the blood. Mann-Whitney test was used for statistical analysis. **p<0.001, ***p<0.0001.

**Figure 9:**
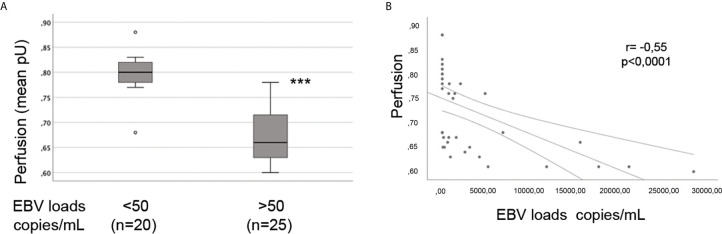
Increased EBV loads inversely correlate with hand perfusion in SSc patients. EBV DNA was extracted from whole blood and quantified by qPCR. EBV quantitative curve was generated using ABI`s Viral load control. qPCR analysis was performed using primers and probe designated to amplified the EBV polymerase BALF5, and measured using SYBR Green chemistry. Shown here are copies of viral DNA calculated by standard curve. **(A)** SSc patients with significantly higher level of EBV DNA loads show reduction in the hand perfusion compared to SSc patients with low or undetectable level of EBV DNA loads. Mann-Whitney test was used for statistical analysis **(B)** Inverse correlation between increase of EBV DNA copies and reduction in blood perfusion in SSc patients (Spearman r=0.55, p<0.0001). ***p<0.0001.

The presence of vascular injury occurs in many tissues, including the skin, in the early phase of SSc (4). Vasospasm, characterized by Raynaud`s phenomenon, and a marked decrease in the number of capillaries in clinically involved and uninvolved skin has been reported, suggesting that endothelial cell death and defective angiogenesis might be responsible for the EC loss and vessel rarefaction in SSc (74). These pathological consequences lead to the presence of ischemic digital ulcers (DUs) with a marked decreased perfusion in the affected organs and the skin of SSc patients (75). Given that EBV induced an IFN response in infected ECs, and IFNs promote EC damage and loss (76), we next interrogated whether SSc patients with elevated peripheral blood EBV loads might present clinical signs of vascular injury. Clinical examination of the number of DUs was analyzed in 41 SSc patients. Remarkably, SSc patients with new active DUs showed significantly higher levels of EBV loads than patients without DUs (**Figure 8A**). Moreover, SSc patients with a past history of digital ulcers show increased levels of viral loads compared to SSc patients with no history of DUs and low or undetectable level of EBV loads (**Figure 8B**). Given that specific capillary abnormalities occur early in the disease and can be detected by nailfold videocapillaroscopy (NVC) (77, 78), 41 SSc patients with diffuse cutaneous disease were analyzed to identify distinct scleroderma-specific patterns. NVC showed that SSc patients with active and late patterns have significantly increased EBV DNA loads, while patients with the early capillaroscopic pattern show a low or undetectable levels of EBV loads in the peripheral blood (**Figure 8C**). Further confirming association of EBV infection with clinical signs of SSc vasculopathy, significant reduction in skin perfusion has been found in patients with higher level of EBV loads compared to SSc patients with undetectable level of viral loads (**Figures 9A, B**). Altogether, these results suggest that lytic EBV antigens with increased virus production associates with clinical signs of vascular injury and altered perfusion in the context of SSc.

## Discussion

Several mechanisms have been described to explain how EBV triggers autoimmune disease, such as antigen cross-reactivity with self-nuclei protein and/or bystander activation of autoreactive cells (34, 39, 79–82). In this study, we report a novel feature employed by EBV that triggers the TLR9 antiviral response and markers of vascular injury in infected endothelial cells. We also demonstrate for the first time that human monocytes bound to EBV recombinant virus are capable to transfer EBV to the endothelial cells, suggesting that circulating EBV infected monocytes, directly and indirectly might contribute to the vascular injury in SSc. While EBV has not previously been associated with monocyte infection, it is notable that the gamma-herpesvirus MHV 68 infects dendritic cells and macrophages (83).

Evidence of EBV infection in ECs has been reported (66, 84). It has been shown that EBV could infect vascular ECs both in human tissues and in cultures. EBER-positive cells have been reported in the ECs from patients with systemic granulomatous arteritis, an autoimmune disease characterized by vasculitis of the large vessels (85). Additional evidence of EBV infecting ECs were reported in primary human-umbilical cord-derived ECs (HUVECs) exposed to EBV-immortalized lymphoblastoid cell lines (LCL). It has been found that ECs, when infected with EBV, express genes related to EBV latency programs, such as EBNA1 and produced high level of IL-6 (66). Further confirmation of EC infectability by EBV comes from a study of multiple sclerosis (MS), another autoimmune disease where EBV has been implicated in the pathogenesis (84). Specifically, it has been shown that EBV infects human brain microvascular ECs (HBMECs) leading to EC activation and increased production of CCL-5 (RANTES) and the adhesion molecule, ICAM-1 in infected cells, suggesting that infected microvascular cells release inflammatory cytokines upon EBV infection (84). Here we report a novel system that successfully infects human dermal microvascular endothelial cells (HDMECs) *in vitro*, providing evidence that EBV is able to infect ECs using monocytes as a vehicle for infection (86, 87). It is likely that EBV uses alternative strategies to infect ECs that bypass the absence of CD21, similar to the described transmission of EBV to human epithelial cells and fibroblasts (41, 52, 88).

An interesting aspect of our study is that we found expression of EBV-BZLF1/ZTA immediate early lytic- (**Figure 5B** and **Figure 6A**) and early lytic BFRF1 genes (data not shown) in infected cells, while EBV/late lytic genes were not detected, suggesting that EBV replication is incomplete, which implies that some but not all lytic genes are expressed in infected cells (89). Although we were not able to determine at what stage the viral cycle becomes abortive, it is possible that viral replication is interrupted at the early stage of the lytic infection, as it has seen in fibroblasts infected with EBV (41). Whether lytic DNA replication, which is driven by EBV early genes and is required for late gene expression, remains to be investigated.

Activation of the TLR9 signaling has been implicated in the pathogenesis of several autoimmune diseases including SSc (90–93). Elevation of TLR9 expression and increased TLR9 signature has been found in the skin of SSc patients compared to control skin (90). Furthermore, recent studies showed the importance of TLR9 activation pathway in inducing pro-fibrotic profile responses, involving autocrine TGF‐β production, in human normal dermal fibroblasts stimulated with the TLR9 ligand CpG (90). Here, in our study, we show that EBV up-regulates TLR9 mRNA and TLR9 innate immune mediators in infected ECs, suggesting that EBV DNA is implicated in the activation of the TLR9 pathway and that viral DNA might be recognized by TLR9 in ECs. Given that the linear form of unmethylated EBV ds-DNA, which is produced by EBV lytic replication can be detected by TLR9, while the methylated EBV DNA, which is abundant in particular latency states remains invisible to TLR9 (94–97), it is possible that activation of TLR9 is induced by un-methylated EBV ds-DNA in infected ECs and that viral nucleic acids might represent the TLR9 ligand in SSc dermal endothelial cells.

In agreement with the previous finding that interferon alpha is up-regulated in SSc ECs, we found that EBV mediates TLR9 inflammatory response by inducing expression of the IRF innate immune mediators and IFN inducible genes MX1 and CXCL10. Since type I IFNs are potent antiangiogenic cytokines known to promote endothelial death and inhibit endothelial migration (76, 98–101), it is conceivable that EBV infection through the activation of the TLR9 innate immune inflammation and type I IFN contributes to the endothelial cell loss and vasculopathy in SSc. Moreover, given that more than one EBV lytic gene can destroy nuclear membranes during lytic infection (62, 102), it is also possible that expression of EBV early lytic genes in infected cells may directly cause EC apoptosis.

We also found that genes such as endothelin 1, thrombospondin 1 and heparan sulfate proteoglycan 2 (HSPG2), which are not known to be activated by TLR9 inflammatory response, are induced by EBV in infected endothelial cells. One explanation could be that TLR9 activates distinct gene profiles depending on the infected cell type, in this case the endothelial cells, or pathways different from TLR9 might be activated by EBV in infected endothelial cells. Given that these genes are of particular interest as they have been associated with vascular activation and dysfunction as well as fibrosis in SSc and several other diseases, further studies might be required to clarify this important aspect.

In this study we report for the first time that EBV DNA load is highly increased in SSc blood and plasma. Although the origin of EBV DNA in the circulation is not clear, it is possible that it may be derived from apoptotic cells as detected at early stage in post-transplant lymphoproliferative disease (PTLD), in patients with nasopharyngeal carcinoma and Hodgkin’s disease (103). It is generally accepted that cells harboring EBV DNA are likely to be B cells (104), but occasionally T cells, natural killer cells, monocytes, and immature dendritic cells can be infected as well (105–107). Consistent with this observation, we report that EBV DNA load is largely located in B cells and circulating monocytes from patients with SSc. The finding that SSc monocytes carry EBV DNA loads is in agreement with our previous observation that circulating monocytes express EBV immediate early, early and late lytic genes (35), suggesting that SSc monocytes might be capable to produce and release virions in SSc.

Although the mechanism responsible for the increase of the EBV DNA load in autoimmune diseases and SSc remains to be established, it is possible that impaired immune function could lead to an increase in EBV DNA replication in SSc patients, rendering these patients long-term viral carriers. Given the numerous HLA polymorphisms in the HLA-class-I/II-genes strongly associating with the risk of developing autoimmune diseases including SSc (2, 59, 108), it is also possible that SSc genetic susceptibility associated with the genetic variability in EBV strain might predispose SSc patients to an uncontrolled, persistent active EBV lytic infection (89, 109–113). An important aspect of this study is that SSc monocytes carry high levels of EBV DNA while monocytes from SLE patients do not. One explanation could be that the presence of pro-fibrotic phenotype that underlies SSc monocytes might facilitate EBV reactivation and/or viral persistence in SSc patients. Given that TGFβ is important to induce SSc pro-fibrotic phenotype and TGFβ is also important for EBV replication, since it induces Zta and could potentially reactivate EBV *in vivo* (36, 114, 115), it possible that pro-fibrotic phenotype promotes EBV reactivation in SSc monocytes.

Our data show that EBV DNA loads are increased in the blood from SSc, with a frequency of 60% in SSc patients. Notably, almost all, with the exception of 4, of the SSc patients enrolled in the study, were not in a state of immunosuppression therapy, indicating that the increase in EBV DNA replication occurs spontaneously and independently of immunosuppressant therapy in SSc.

Our findings that SSc with high EBV DNA copies are associated with severe clinical signs of vascular injury, while SSc patients with lower or undetectable levels of EBV DNA show milder or no signs of vessel damage, suggest that EBV loads alone might represent a useful tool for monitoring vascular damage in SSc. As the few patients that showed lower or undetectable level of circulating EBV DNA loads did not have signs of active vascular injury, it is possible that those patients with lower viral DNA show less risk to develop vascular diseases. As an alternative explanation, it could be that variability in EBV DNA loads might reflect different organ involvement or represent different stage of the disease in SSc. Noteworthy, we observed that four SSc patients with highly increased viral loads and severe signs of vascular injury were affected by pulmonary arterial hypertension (PAH) and SSc renal crisis (three and one SSc patients, respectively), suggesting that the vascular system of these organs might also be a target of EBV infection. Therefore, monitoring for dynamic changes in EBV loads might be important in identifying those at risk for developing vascular disease and provides more relevant information for adapting therapy. Further studies will be required to evaluate this important aspect.

A limitation of the current study is that it was not possible to correlate viral load in monocytes with clinical signs of vascular injury. For this retrospective study, much clinical data was not available for the SSc patients with increased EBV viral loads in monocytes. Future studies involving evaluation of EBV load in monocytes from SSc patients, with and without vascular damage, may provide further evidence of the role of EBV as a crucial co-factor for the development of SSc vasculopathy.

In summary, microvascular injury occurs in the early stage of SSc, and widespread change of the microvasculature is a cardinal feature of SSc. Thus, understanding the endothelial cell injury induced by lytic EBV has the potential to address the concept that an active viral infection drives endothelial cells dysfunction and vessel injury in SSc. In addition, our data point to up-regulation of EBV DNA loads as a potential biomarker for developing vascular injury in SSc. Our results provide the framework to support the development and testing of antiviral therapeutic interventions in SSc treatment paradigms.

## Data Availability Statement

The original contributions presented in the study are included in the article/**Supplementary Material**. Further inquiries can be directed to the corresponding author.

## Ethics Statement

The studies involving human participants were reviewed and approved by Boston University IRB committee and Sapienza University ethic committee. The patients/participants provided their written informed consent to participate in this study.

## Author Contributions

All authors participated in the preparation of the manuscript in a significant way. Study design: GF, AF, ER BG. Acquisition of clinical specimens: ER, MY. Experiments performed: AF, ER, GF. Analysis and interpretation data: AF, ER, BG, GF. Statistical analysis: ER, GF. Manuscript preparation: AF, ER, BG, MT, GF. All authors contributed to the article and approved the submitted version.

## Funding

This study was supported by: Scleroderma Foundation Established Investigator Grant” to GF. BG is supported by a Burroughs Wellcome Career Award in Medical Sciences.

## Conflict of Interest

The authors declare that the research was conducted in the absence of any commercial or financial relationships that could be construed as a potential conflict of interest.
