# Supplementary material for: Innate Immune Modulation Induced by EBV Lytic Infection Promotes Endothelial Cell Inflammation and Vascular Injury in Scleroderma
Source: Front Immunol. 2021 Apr 19;12:651013. doi: 10.3389/fimmu.2021.651013 (PMC8089375; doi:10.3389/fimmu.2021.651013)
Supplement: Supplementary file 1 [file Table_1.docx]

**Table S1: Clinical and demographic data of SSc, SLE and healthy donor subjects included in these studies.**

| **Cohort Demographic and Clinical Data** | | |
| --- | --- | --- |
| **Healthy Donors blood samples (n=55)**  **(Boston University cohort n=10) (Sapienza University cohort n=45)** | | |
|  | Age (average, range) | 42.5 (21-65) |
| **SSc blood samples (n=65) (BU cohort n=15) (Sapienza University cohort n=50)** | | |
|  | Age (average, range) | 48.2 (22-71) |
|  | Disease duration, years (average, range) | 6.2 (1-30) |
|  | Lung fibrosis, n (%) | 33/55 (60%) |
|  | Modified Rodnan skin score (average, range) | 24.3 (2-50) |
|  | Digital ulcers, n (%) | 18/41 (44%) |
| Medications | | |
|  | Immunosuppressant, n (%) | 4/65 (6,1%) |
|  | Anti-Endothelin Rec /Vasodilators, n (%) | 33/45 (73%) |
|  | Untreated, n (%) | 28/65 (43%) |
| **SLE blood samples (n=10) (Sapienza University cohort)** | | |
|  | Age (average, range) | 38.1 (20-55) |
|  | Skin lesion, n (%) | 4/10 (40%) |
| Medications | | |
|  | Immunosuppressant, n (%) | none |
|  | Untreated, n (%) | 8/10 (80%) |
| **Healthy Donor skin biopsies (n=10) (Boston University cohort)** | | |
|  | Age (average, range) | 48 (24-66) |
| **SSc skin biopsies (n=10) (Boston University cohort)** | | |
|  | Age (average, range) | 53 (20-76) |
|  | Disease duration, years (average, range) | 3.6 (0.8-6.5) |
|  | Lung fibrosis, n (%) | **NA** |
|  | Modified Rodnan skin score (average, range) | 28,4 (12-50) |
|  | Digital ulcers, n (%) | **NA** |
| Medications | | |
|  | Immunosuppressant, n (%) | 0/10 |
|  | Anti-Endothelin Rec /Vasodilators, n (%) | **NA** |
|  | Untreated, n (%) | 8/10 (80%) |
